# Mastery Learning Ensures Correct Personal Protective Equipment Use in Simulated Clinical Encounters of COVID-19

**DOI:** 10.5811/westjem.2020.6.48132

**Published:** 2020-07-21

**Authors:** Nicholas Pokrajac, Kimberly Schertzer, Cori M. Poffenberger, Al’ai Alvarez, Paloma Marin-Nevarez, Christopher Winstead-Derlega, Michael A. Gisondi

**Affiliations:** Stanford University, Department of Emergency Medicine, Palo Alto, California

## Abstract

**Introduction:**

The correct use of personal protective equipment (PPE) limits transmission of serious communicable diseases to healthcare workers, which is critically important in the era of coronavirus disease 2019 (COVID-19). However, prior studies illustrated that healthcare workers frequently err during application and removal of PPE. The goal of this study was to determine whether a simulation-based, mastery learning intervention with deliberate practice improves correct use of PPE by physicians during a simulated clinical encounter with a COVID-19 patient.

**Methods:**

This was a pretest-posttest study performed in the emergency department at a large, academic tertiary care hospital between March 31–April 8, 2020. A total of 117 subjects participated, including 56 faculty members and 61 resident physicians. Prior to the intervention, all participants received institution-mandated education on PPE use via an online video and supplemental materials. Participants completed a pretest skills assessment using a 21-item checklist of steps to correctly don and doff PPE. Participants were expected to meet a minimum passing score (MPS) of 100%, determined by an expert panel using the Mastery Angoff and Patient Safety standard-setting techniques. Participants that met the MPS on pretest were exempt from the educational intervention. Testing occurred before and after an in-person demonstration of proper donning and doffing techniques and 20 minutes of deliberate practice. The primary outcome was a change in assessment scores of correct PPE use following our educational intervention. Secondary outcomes included differences in performance scores between faculty members and resident physicians, and differences in performance during donning vs doffing sequences.

**Results:**

All participants had a mean pretest score of 73.1% (95% confidence interval [CI], 70.9–75.3%). Faculty member and resident pretest scores were similar (75.1% vs 71.3%, p = 0.082). Mean pretest doffing scores were lower than donning scores across all participants (65.8% vs 82.8%, p<0.001). Participant scores increased 26.9% (95% CI of the difference 24.7–29.1%, p<0.001) following our educational intervention resulting in all participants meeting the MPS of 100%.

**Conclusion:**

A mastery learning intervention with deliberate practice ensured the correct use of PPE by physician subjects in a simulated clinical encounter of a COVID-19 patient. Further study of translational outcomes is needed.

## INTRODUCTION

The pandemic caused by severe acute respiratory syndrome coronavirus 2 (SARS-CoV2) and its resultant clinical illness, coronavirus disease 2019 (COVID-19), has stressed healthcare systems across the world. Nearly 10,000 healthcare workers contracted COVID-19 in the United States (US) alone during the period from February 12–April 9, 2020.^1^ SARS-CoV2 spreads by means of surface contamination, exposure to droplets containing viral particles, and through aerosolization, particularly during high-risk procedures.^2^ Healthcare workers are at increased risk for infection given frequent exposure to the virus during routine patient care.

Proper use of personal protective equipment (PPE) by healthcare workers is well established to decrease the rate of infectious disease transmission, including by means of self-contamination.^3^ However, prior studies have demonstrated that healthcare workers are inconsistent in the proper use of PPE. Contamination rates during donning and doffing of PPE range from 10–100%.^3^,^4^ Deviations from accepted protocols for donning and doffing PPE also occur in 50–87% of healthcare workers.^4^–^6^ Therefore, effective education to improve donning, wearing, and doffing of PPE is critical for healthcare worker safety.

A number of educational interventions to improve correct use of PPE have been performed with varying success.^3^,^7^ A growing body of evidence suggests that simulation-based mastery learning (SBML) is more effective than other educational techniques to attain procedural skill mastery.^8^,^9^ SBML is an educational technique that must include the following: 1) baseline testing of a target skill; 2) discrete learning objectives organized by rising difficulty; 3) attentive learner engagement during the activity; 4) a defined minimum passing standard (MPS); 5) testing during the educational process to direct learning and evaluate achievement of the MPS; 6) advancement after reaching the MPS; and 7) continued practice until the MPS is achieved.^10^ This method often is paired with deliberate practice, which requires highly motivated students to engage in focused, repetitive practice toward a specified goal with informative feedback to correct errors. The goal of SBML is to have all participants achieve an expert level of skill with minimal to no variation, which is crucial in patient care environments. Moreover, implementation of a SBML curriculum may improve translational outcomes.^11^–^13^ The goal of this study was to determine whether mastery learning methodology can improve physician ability to correctly don and doff PPE during a simulated clinical encounter with a COVID-19 patient.

## METHODS

### Study Design and Approval

Physician subjects participated in a mastery learning educational intervention with a simulated clinical encounter of a patient with COVID-19.^14^ Prior to the intervention, all participants had received institution-mandated N95 mask-fit testing and training on the proper use of PPE via an online video and a supplemental online infographic demonstrating steps of donning and doffing of PPE. Participants were assessed on their ability to correctly don and doff PPE using a checklist before and after the intervention on the same day. The study was approved by our local institutional review board (IRB #55851).

### Participants and Study Setting

Participants included clinical faculty members and emergency medicine (EM) resident physicians in the ED at a large, academic tertiary care center from March 31–April 8, 2020. The assessment was conducted in an administrative office space designed to simulate a medical examination room with a door and no anteroom, with a patient under airborne, droplet, and contact precautions.

### Outcome Measures and Measuring Instrument

**Figure 1:**
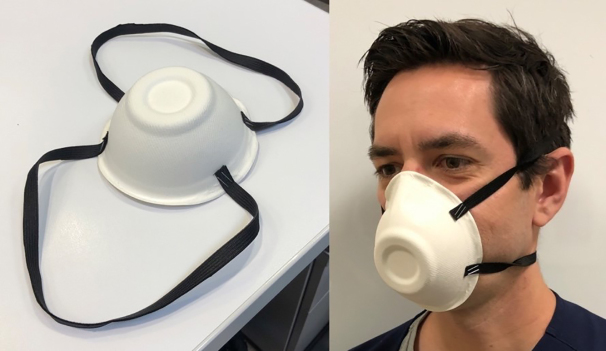
A simple substitution of an N95 facemask for simulated patient encounters.

Participants were assessed individually prior to the intervention using a 21-item checklist of steps for donning and doffing PPE using a double-glove technique (Online Supplement).^15^,^16^ The checklist was developed by one author and adapted from existing best-practices guidelines on PPE use from the US Centers for Disease Control and Prevention (Atlanta, GA) and Stanford University (Palo Alto, CA,).^15^ Additional authors with expertise in PPE use, medical education, and checklist design reviewed and modified the checklist. The checklist underwent final review and approval by infection control specialists at our institution to ensure completeness, compliance, and internal consistency within the hospital system. Equipment consisted of Medline isolation gowns, Medline Fitguard nitrile exam gloves (Medline Industries, Inc, Northfield, IL), and DeRoyal SPEyes Eye ShieldZ (DeRoyal Industries, Powell, TN). Due to a national shortage of N95 face masks at the time of this project, a simple substitution of quarter-inch elastic bands stapled to an 8-ounce paper bowl was used (Figure 1).

Adherence to checklist items during testing was assessed by four reviewers. All reviewers underwent a one-hour training on the use of the checklist as a rating instrument, which consisted of checklist review, demonstration of correct PPE use by a study author, deliberate practice, and mock assessments. Reviewers were instructed to give zero points to items not done or performed incorrectly and one point to items performed correctly. All reviewers independently scored at least 10% of participants to determine inter-rater reliability of the instrument.

A MPS for correct completion of checklist items was determined by 16 experts using a combination of Mastery Angoff and Patient Safety approaches.^17^–^19^ All 16 experts were EM clinical faculty members, 10 of whom have advanced training in medical education, three in medical simulation, and one in emergency medical services.

### Educational Intervention

Physician subjects individually participated in a mastery learning educational intervention if they did not achieve the MPS on the pretest assessment. The intervention consisted of an in-person expert demonstration of proper donning and doffing of PPE using the 21 steps outlined in the checklist, followed by a 20-minute opportunity for deliberate practice with feedback. If participants again did not achieve the MPS on reassessment, they were given additional opportunities for deliberate practice until the MPS was achieved. Final scores were determined by reviewers unblinded to initial participant assessments.

### Statistical Analysis

We performed statistical analysis using SPSS Statistics for Windows, Version 24, (IBM Corp., Armonk, NY). Inter-rater reliability was determined by calculating Cohen’s kappa statistic. We used a two-tailed paired T-test to compare pre-and posttest scores. The difference between faculty and resident physicians’ scores was calculated using a two-tailed Student’s T-test.

## RESULTS

A total of 117 physician subjects participated in the study, including 56 faculty members (56/88, 63.6%) and 61 EM resident physicians (61/62, 98.3%). Participant demographic information is summarized in Table 1.

Standard setting using a Patient Safety approach resulted in 19/21 of the checklist items deemed critical for safety. A Mastery Angoff score calculated for the two non-critical items was 90.2%. Requiring completion of all items deemed critical from the Patient Safety approach plus 90.2% of two non-critical items resulted in the final MPS set at 100%.^17^–^19^

**Figure 2:**
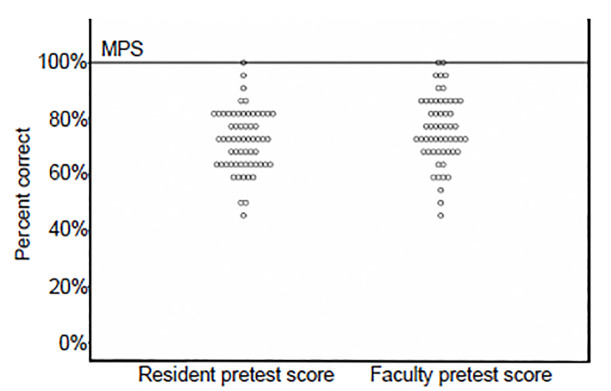
Comparison of resident vs faculty member pretest scores. *MPS*, minimum passing score.

Agreement between assessors across checklist items ranged from moderate to strong (κ = 0.70 to 0.87). Two faculty members (3.6%) and one resident physician (1.6%) successfully achieved the MPS on pre-intervention assessment. Mean pretest score among all participants was 73.1% (95% confidence interval [CI], 70.9–75.3%). There was no significant difference between the mean pretest scores of faculty members and resident physicians (75.1% vs 71.3%, p = 0.082) (Figure 2).

Mean pretest doffing scores were significantly lower than donning scores (65.8% vs 82.8%, p<0.001). The items most commonly not completed or incorrectly completed included “adjusts nosepiece of mask,”, “demonstrates mask seal check,” “doffs eye shield in room,” “disposes of eye shield in room,” and “performs hand hygiene on inner gloves” (Table 2).

**Figure 3:**
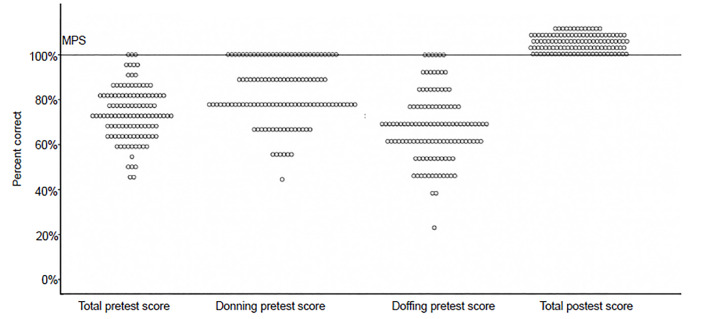
Comparison of pretest and posttest scores across all participants. *MPS*, minimum passing score.

After our educational intervention, the mean participant score increased 26.9% (95% CI of the difference 24.7–29.1%, p<0.001) (Figure 3). No participants required more than 20 minutes to achieve mastery.

## DISCUSSION

This study demonstrated that a (SBML) intervention with deliberate practice led to significant improvement in both faculty and resident physicians’ ability to correctly don and doff PPE. On pretest assessment, faculty and resident participants demonstrated frequent errors during donning and doffing of PPE despite completing comprehensive, institution-mandated online training. Similar to prior studies, errors were more common during doffing of protective equipment, which is when providers are at greatest risk of self-contamination.^20^ Therefore, these results highlight a critical role for SBML to improve correct PPE use and suggest that the sole utilization of online modules for PPE use may be inadequate for workplace safety.

SBML is a highly effective method of teaching procedures.^21^ The focus of SBML is the achievement of a fixed learning outcome; training time varies between participants to allow adequate opportunities for deliberate practice with feedback. While completion time varied, no participant in our study exceeded 20 minutes of training time and all participants met our predetermined learning outcome on post-intervention testing. This approach is in contrast to more common instructional techniques, in which teaching time is fixed and participant achievement varies. As such, rigorous adherence to SBML principles as used in this study likely represents the gold standard for procedural training.^13^

Previous research demonstrated SBML to be an effective method of teaching both invasive and non-invasive procedures, including lumbar puncture,^9^ central line insertion,^11^,^12^ paracentesis,^22^ and thoracentesis,^8^,^23^ among others.^13^ Mastery learning also achieves translational outcomes that result in better patient care (T3) as defined by lower complications rates during high-risk procedures.^11^,^12^,^23^ The proper donning and doffing of PPE for aerosolized infection is similarly high risk and correct completion (fixed achievement) of the don/doff sequence is absolutely necessary to minimize risk to patients and providers.

We designed our mastery learning intervention to simulate patient care performed in locations without anterooms, which comprise the majority of hospital and outpatient clinical spaces. This allows for authenticity in the logistical challenges present when caring for a high frequency of COVID-19 patients requiring airborne, droplet, and contact precautions. In addition, double-glove technique mitigates skin contact with potential surface contaminants while exiting a patient’s room. Providers may also have improved hand comfort with the application of alcohol-based gels over inner gloves rather than skin, given the frequency of required hand sanitization during donning and doffing of PPE.^24^ The double-glove technique is presumed to increase provider safety for a variety of infectious diseases beyond COVID-19.

Finally, our intervention standardized PPE use among our physicians by allowing participants the opportunity to ask nuanced questions and practice repeatedly. This supportive and psychologically safe training may be especially important to mitigate provider anxiety in anticipation of a COVID-19 surge. A shared mental model also allows observation and direct feedback by faculty-resident pairs during donning and doffing of PPE. The ability to train the majority of our emergency physicians in a one-week time period suggests that comprehensive physician training is feasible. Future study is needed to determine the potential translational impact of our intervention.

## LIMITATIONS

This study had several limitations. First, the checklist instrument required use of a double-glove rather than single-glove technique, the latter of which was included as part of the pre-existing, institution-mandated online training for PPE use. As a result, providers may have been less familiar with the double-glove technique on pretest. Double-glove technique was chosen due to the prevalence of patient care areas without anterooms at the institution. This technique was also chosen in an effort to optimize both comfort and personal protection in light of changing guidelines during the pandemic. Second, the strict MPS of the assessment may have decreased the initial pass rate on pretest. Third, reviewers were unblinded to pretest results, which may have influenced posttest scores. Fourth, due to time constraints, participants completed repeat testing immediately following deliberate practice, which limited assessment for skill retention. Finally, while previous studies demonstrated improvements in translational outcomes following SBML, it is still unclear whether this intervention will result in an observable change of behavior during patient care.

## CONCLUSION

Mastery learning methodology with deliberate practice is an effective and feasible educational modality for training a large number of physicians in the proper use of PPE in simulated clinical encounters of patients with COVID-19. Before our training intervention, very few providers passed a rigorous assessment of PPE donning and doffing despite institution-mandated, online PPE training. After undergoing the SBML intervention, all participants successfully completed assessment with 100% accuracy. In addition, the double-glove technique may offer additional provider protection when caring for a high volume of COVID-19 patients in treatment areas that lack anterooms. Further study of translational outcomes resulting from our intervention is needed.

## Supplementary Information



## Figures and Tables

**Table 1:** Demographic information of participants in a simulation-based mastery learning course with deliberate practice to improve use of personal protective equipment.

	Participants (%)(N=117)
Professoriate rank of faculty members	
Professor	7 (6.0)
Associate	13 (11.1)
Assistant	28 (23.9)
Instructor	8 (6.8)
Total faculty members	56 (47.9)
Postgraduate year (PGY) of resident physicians	
PGY4	15 (12.8)
PGY3	15 (12.8)
PGY2	14 (12.0)
PGY1	16 (13.7)
Total resident physicians	61 (52.1)

**Table 2:** Mastery Learning Checklist* for donning and doffing personal protective equipment, with pre-intervention checklist performance by item and participant role.

Items not completed	Faculty members (N = 56)	Residents (N = 61)	Total participants (N = 117)
Donning sequence			
Performs hand hygiene	11 (19.6)	19 (31.1)	30 (25.6)
Dons inner layer of gloves	2 (3.6)	3 (4.9)	5 (4.3)
Dons gown	9 (16.1)	3 (4.9)	12 (10.3)
Dons mask	1 (1.8)	5 (8.2)	6 (5.1)
Adjusts nosepiece of mask	14 (25)	28 (45.9)	42 (35.9)
Demonstrates mask seal check	29 (51.8)	48 (78.7)	77 (65.8)
Dons eye shield	2 (3.6)	0 (0.0)	2 (1.7)
Dons outer layer of gloves	3 (5.4)	4 (6.6)	7 (6.0)
Enters room and closes door	0 (0.0)	0 (0.0)	0 (0.0)
Doffing sequence			
Begins doffing 6′ from patient	7 (12.5)	3 (4.9)	10 (8.5)
Doffs gown with outer gloves	15 (26.8)	14 (23.0)	29 (24.8)
Disposes of gown and outer gloves in room	3 (5.4)	3 (4.9)	6 (5.1)
Performs hand hygiene on inner gloves	25 (44.6)	25 (41.0)	50 (42.7)
Doffs eye shield in room	30 (53.6)	42 (68.9)	72 (61.5)
Disposes of eye shield in room	30 (53.6)	43 (70.5)	73 (62.4)
Performs hand hygiene on inner gloves	43 (76.8)	52 (85.2)	95 (81.2)
Exits room and closes door	2 (3.6)	3 (4.9)	5 (4.3)
Performs hand hygiene on inner gloves	41 (73.2)	44 (72.1)	85 (72.6)
Removes and disposes of mask	4 (7.1)	9 (14.8)	13 (11.1)
Removes and disposes of gloves	20 (35.7)	19 (31.1)	39 (33.3)
Performs hand hygiene	2 (3.6)	1 (1.6)	3 (2.6)

* Two-glove technique for personal protective equipment use with airborne, contact, and droplet precautions. See Online Supplement 1 for definitions of “complete” for each checklist item.
