# Supplementary material for: Mastery Learning Ensures Correct Personal Protective Equipment Use in Simulated Clinical Encounters of COVID-19
Source: West J Emerg Med. 2020 Jul 21;21(5):1089–94. doi: 10.5811/westjem.2020.6.48132 (PMC7514383; doi:10.5811/westjem.2020.6.48132)
Supplement: Supplementary file 1 [file wjem-21-1089-s001.docx]

**Donning and Doffing of Personal Protective Equipment (PPE) in Patient Care Areas without an Anteroom**

**Stanford University Department of Emergency Medicine Mastery Learning Checklist**

|  |  | 0 | 1 |
| --- | --- | --- | --- |
| **Donning Sequence** | | | |
| 1 | *Performs hand hygiene* | Not done or done incorrectly | Done correctly  (Cleans all areas of both hands for at least 20 seconds.)  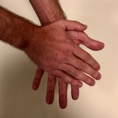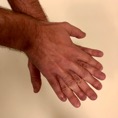  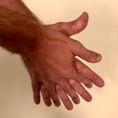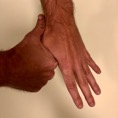  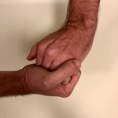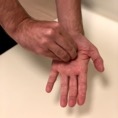 |
| 2 | *Dons inner layer of gloves* | Not done | Done |
| 3 | *Dons gown* | Not done or done incorrectly | Done correctly  (Gown wrapped across back, providing full coverage, tied in front.) |
| 4 | *Dons mask* | Not done or done incorrectly | Done correctly  (Top band rests above ear on crown of head, bottom band rests below ear on nape of neck.) |
| 5 | *Adjusts nosepiece of mask* | Not done or done incorrectly | Done correctly  (Using both hands, mold nose strip to shape of nose.) |
| 6 | *Demonstrates mask seal check* | Not done or done incorrectly | Done correctly  (Place hands over facemask, covering as much surface area as possible, inhale and exhale. Mask should slightly bulge during exhale or slightly collapse during inhale.) |
| 7 | *Dons eye shield* | Not done | Done |
| 8 | *Dons outer layer of gloves* | Not done or done incorrectly | Done correctly  (No skin should be showing between glove and gown cuffs.) |
| 9 | *Enters room and closes door* | Not done | Done |
| **Doffing Sequence** | | | |
| 1 | *Begins doffing at least 6’ from patient* | Not done | Done |
| 2 | *Doffs gown with outer gloves* | Not done or done incorrectly | Done correctly  (Breaks tie, removes gown from top down, inverts gown and sleeves, removes gown and outer glove with inverted portion.) |
| 3 | *Disposes of gown and outer gloves*  *in room* | Not done or done incorrectly | Done correctly  (Rolls gown up away from body, uses biohazard bin, does not compress into trash.) |
| 4 | *Performs hand hygiene on inner gloves* | Not done or done incorrectly | Done correctly  (Cleans all surfaces of both hands for at least 20 seconds. May verbalize this step.) |
| 5 | *Doffs eye shield in room* | Not done or done incorrectly | Done correctly  (Grabs from side of shield, must not touch face.) |
| 6 | *Disposes of eye shield in room* | Not done or done incorrectly | Done correctly  (Does not compress into trash.) |
| 7 | *Performs hand hygiene on inner gloves* | Not done or done incorrectly | Done correctly  (Cleans all surfaces of both hands for at least 20 seconds. May verbalize this step.) |
| 8 | *Exits room and closes door* | Not done | Done |
| 9 | *Performs hand hygiene on inner gloves* | Not done or done incorrectly | Done correctly  (Cleans all surfaces of both hands for at least 20 seconds. May verbalize this step.) |
| 10 | *Doffs and disposes of mask* | Not done or done incorrectly | Done correctly  (Leans over trash bin, removes bottom band, then top band, does not compress into trash.) |
| 11 | *Doffs and disposes of gloves* | Not done | Done correctly  (Removes one glove without touching skin, removes the second by inserting finger under wrist of glove without touching surface of glove.) |
| 12 | *Performs hand hygiene* | Not done or done incorrectly | Done correctly  (Cleans all surfaces of both hands for at least 20 seconds. May verbalize this step.) |

All items in doffing phase must be done in correct sequence.

Participant fails if touches mask or eye shield in patient room, or if they reach under gown.
